# Pancreatoduodenectomy with or without Pyloric Preservation: A Clinical Outcomes Comparison

**DOI:** 10.1155/2008/719459

**Published:** 2009-01-29

**Authors:** Sean P. Dineen, Christina L. Roland, Roderich E. Schwarz

**Affiliations:** Division of Surgical Oncology, Department of Surgery, University of Texas (UT) Southwestern Medical Center, Dallas, TX 75390, USA

## Abstract

Pyloric preservation (PP) can frequently be performed at the time of pancreatoduodenectomy (PD), although some reports have linked it to inferior outcomes such as delayed gastric emptying (DGE). We reviewed records in a single-surgeon practice to assess outcomes after PD with or without PP. There were 133 PDs with 67 PPPDs and 66 PDs. Differences between PPPD and PD groups included cancer frequency, tumor size, OR time, blood loss, and transfusion rate. However, postoperative morbidity rate and grade, NG tube duration, NGT reinsertion rate, DGE, and length of stay were similar. There was no difference among patients with pancreatic cancer. No detrimental outcomes are associated with pyloric preservation during PD. Greater intraoperative ease and superior survival in the PPPD group are due to confounding, tumor-related variables in this nonrandomized comparison. Nevertheless, we intend to continue the use of PP with our technique in patients who meet the stated criteria.

## 1. BACKGROUND

Pancreatic cancer continues to carry a dismal prognosis with a 5-year survival of approximately five percent [1]. For patients that present with resectable disease, pancreatoduodenectomy (PD) offers the best chance for cure among otherwise poor treatment options [2]. The standard PD, or Whipple-Kausch operation, involves resection of the head of the pancreas, duodenum, common bile duct, gallbladder, and distal stomach including the pylorus. A gastrojejunostomy restores GI continuity to the stomach. However, there is debate regarding the necessary extent of resection. In the 1970s, Traverso and Longmire popularized a pylorus-preserving pancreatoduodenectomy, initially for chronic pancreatitis [3]; interestingly, both initial PDs by Kausch in 1909 and Whipple in 1934 involved preservation of the antrum and pylorus. A pylorus-preserving PD (PPPD) is similar to the “classic” or standard PD except that the distal stomach and pylorus are left intact and continuity is restored through a duodenojejunostomy. The rationale for this modification is that it may allow for normal long-term gastric function, with a controlled release of gastric contents and a reduced gastric accumulation of small bowel succus. However, there are some reports indicating that this technique may generate postoperative challenges due to delayed gastric emptying (DGE) [4–7]. Additionally, there are concerns about the adequacy of margins for cancer operations in which the pylorus is preserved [8]. Prospective randomized trials [6, 9–11], three meta-analyses [8, 12, 13], and an extensive literature review [7] have not led to uniformly congruent or firm conclusions whether PPPD has beneficial or adverse effects, although most larger trials and the more recent pooled analyses have failed to show that an increased frequency of DGE is clearly associated with PPPD. The reasons for a lack of clear interpretability of outcome differences between PD and PPPD in some randomized controlled trials are lack of power and confounding factors affecting the outcomes of interest. For instance, Lin et al. randomized 36 patients and were able to compare 19 PDs to 14 PPPDs; the only obvious difference was a delayed gastric emptying in 1 of 19 PD patients, and 6 of 14 individuals after PPPD [14]. Yeo et al. performed a large, single institution trial of 146 “standard” locoregional pancreatic head resections (with an 86% PPPD rate), compared to 148 radical PDs without PP, but with extended lymphadenectomy and perivascular mesenteric soft tissue dissection; the groups were well balanced regarding tumor type, stage, and operative findings, but the resulting complication rate, delayed gastric emptying, and hospital stay were all significantly higher in the radical dissection group and therefore not attributable to any PPPD per se [10]. The purpose of the current study was to analyze our clinical experience with PPPD compared to standard PD based on indications and clinical outcomes, as all procedures followed the same regional and retroperitoneal soft tissue dissection strategy, and the PP with subsequent duodenal anastomosis was the only different component between the two techniques.

## 2. PATIENTS AND METHODS

Clinical, operative, and pathologic information had been prospectively collected. The database contains information from patients treated by a single surgeon in an academic tertiary care practice setting. Patients were included over a ten-year period from 1997 to 2007. All 133 individuals presented with either a head of pancreas or other periampullary malignancy that was biopsy proven, or had suspicious findings for malignancy within a mass lesion, complex cystic lesion, or ductal stricture affecting the periampullary tissues. All patients had undergone preoperative clinical and imaging evaluation; the latter included computed tomography for all, and magnetic resonance imaging, endoscopic ultrasound, or endoscopic retrograde cholangiopancreatography as deemed indicated. Based on this preoperative evaluation, all patients were considered suitable candidates for partial pancreatoduodenectomy. Preoperative biliary decompression for jaundiced patients was not routinely requested, but was in place in the majority of patients prior to any surgical consultation or operative planning.

**Figure 1:**
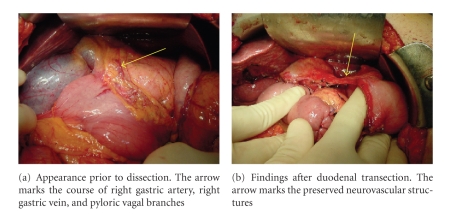
Preservation of the right gastric vasculature and pyloric vagal innervation.

**Figure 2:**
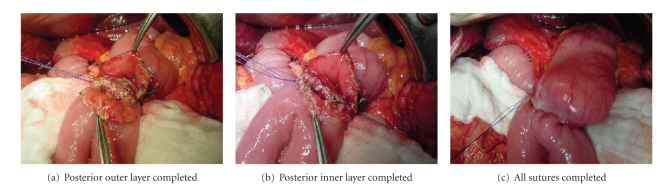
Antecolic duodenojejunostomy, hand-sutured dual-layer technique.

PPPD was performed whenever deemed safe and feasible, based on the intraoperative assessment. This included examination of the mobility of the pyloric ring to exclude inflammatory or neoplastic involvement with the resection specimen. The presence of suspicious perigastric lymph nodes, prohibitive for a PPPD, was examined. Normal anatomy of the distal stomach was ascertained, and patients with known preoperative gastric motility disorders were also excluded from PPPD. Finally, an assessment of whether the pyloric vagal innervation alongside the right gastric vasculature could be preserved was made prior to committing to a PPPD, as sacrificing this structure may contribute to delayed gastric emptying. The technique we employ for PPPD is modified in a sense that the right gastric artery is preserved whenever possible, and regarding the consistent use of our preferred anastomotic technique. Figure 1 illustrates the distal stomach and pylorus of a typical patient and the tissue bridge that remains after the resection described. Figure 2 shows the duodenojejunostomy, which in every case was a hand-sewn, dual layer continuous anastomosis in antecolic position. All gastrojejunostomies in the PD group were equally furbished in antecolic position. The management practice with nasogastric tubes (NGTs) changed during the study interval; during the first five years, NGTs were routinely utilized, and usually removed on postoperative day 1 or 2, based on the amount of gastric aspirate observed; during the later half, an orogastric tube was used intraoperatively, and this was routinely removed at the time of endotracheal extubation. This practice did not differ between PPPD and PD patients. Similarly, placement of a jejunostomy took place routinely as described [15]; its postoperative use in hospital and occasional use after discharge did not differ between the groups.

The definition of DGE varies among authors, as reviewed in detail before [16]; in this study, simple NGT reinsertion need did not automatically qualify for a diagnosis of delayed gastric emptying. We determined a patient to have DGE if there was nausea and vomiting requiring NGT reinsertion for longer than 7 days combined with the inability to take oral nutrition or hydration by postoperative day 10, or if the inability to tolerate oral intake prolonged the patient's hospital stay by more than 2 days. Patients requiring NGT placement during critical care support in the intensive care unit were not included in this group, unless NGT drainage exceeded 1200 mL per 24 hours for greater than three days. Patients with DGE were also classified according to the international study group definition [16]. All patients with postoperative NGT reinsertion did undergo an additional, separate analysis. Postoperative complications were graded according to the 5-grade scale proposed by DeOliveira et al. [17]. Pancreatic leak of fistula formation was graded according to the international study group definition [18]. Postoperative lethal events included those occurring during the in-hospital stay or within 30 days after the procedure, whichever came last.

Continuous data between the PD and PPPD groups were compared via student's *t*-test or Mann-Whitney analysis, based on the original data distribution. Chi square contingency testing was employed for categorical data. Survival data were analyzed with nonparametric Kaplan-Meier statistics; for group differences, a log-rank test was performed. Length of NGT duration and length of hospital stay comparison involved a nonparametric product-limit method as utilized earlier [19]. In-hospital deaths were excluded from this analysis, and group comparisons were performed with a Peto-Peto-Wilcoxon test. All calculations were performed using StatView software for Macintosh, version 5.0.1 (SAS Institute Inc., Cary, NC). Statistical significance of group differences was assumed at a *P* value of <.05.

## 3. RESULTS

### 3.1. Patient demographics

Between 1997 and 2007, 133 of 184 pancreatic resections involved a PD (72%); total pancreatectomies were not included in this analysis. There were 78 women (59%) and 55 men (41%), with a median age of 66 years (range: 38–87). The underlying disease mechanisms providing an indication for resection included 110 malignant processes (83%) and 23 benign disorders (17%). PPPDs (*n* = 67) and PDs without PP (*n* = 66) were numerically balanced. Aside from a slight difference in the frequency of a malignant diagnosis and the primary tumor size, PPPD and PD groups were comparable regarding demographic and clinicopathologic parameters (Table 1). Although the distribution of cancer types did not differ between the two groups in a statistically significant way, the higher percentage of pancreatic primaries and a greater number of cancers in general in the PD group reflect greater oncologic challenges here than those in patients undergoing PPPD.

### 3.2. Operative treatment

The median total operation time for the entire cohort was 6.4 hours, the median estimated blood loss 527 mL, and the overall red blood cell transfusion rate 23%. Several intraoperative parameters differed between the PPPD and PD groups. This included total operative time, estimated blood loss, the amount of intravenous fluid administered, and the blood transfusion rate (Table 2). In all these categories, numbers were more favorable in the PPPD group. However, there were no differences regarding number of units of packed red blood cells transfused per patient transfused, or in the intraoperative urine production.

### 3.3. Postoperative in-hospital outcome

**Figure 3:**
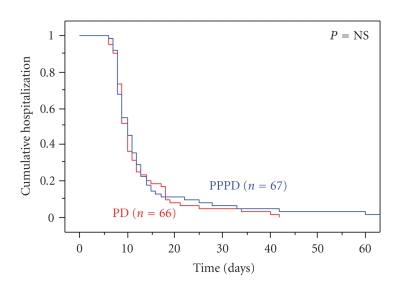
Length of hospital stay, by resection group.

The postoperative overall complication rate was 39%, with a 14% rate of grade 3 or greater severity that required either interventional radiologic, operative, or intensive care management. Fifteen patients experienced a postoperative pancreatic or biliary leak (11%); the leak severity included grade A (*n* = 3, 20%), grade B (*n* = 3, 20%), and grade C (*n* = 9, 60%). There were six postoperative lethal events, for an overall mortality rate of 4.5%. As shown in Table 3, there were no obvious group differences in postoperative morbidity, leak rate or mortality. The median NGT duration for all patients was 1 day. In 19% of patients, the NGT had to be replaced at least once during the postoperative course. However, DGE as defined was observed in only two patients after PPPD, and in three patients past PD. DGE according to the international study group definition included was observed in eight patients (6%), and included grades A (*n* = 5), B (*n* = 1), and C (*n* = 2). Two of the individuals with DGE had a grade B pancreatic leak. In none of these parameters were differences detected between the PD and PPPD groups (Table 3). One patient with DGE after PPPD had a biliary leak, the other had no other identifiable accompanying morbidity event. The median length of stay was 10 days overall. As depicted in Figure 3, the cumulative length of stay comparison did not reveal significant differences between the treatment groups, and the subset of patients with a prolonged hospital stay was similar in the PD and PPPD cohorts.

### 3.4. Survival

**Figure 4:**
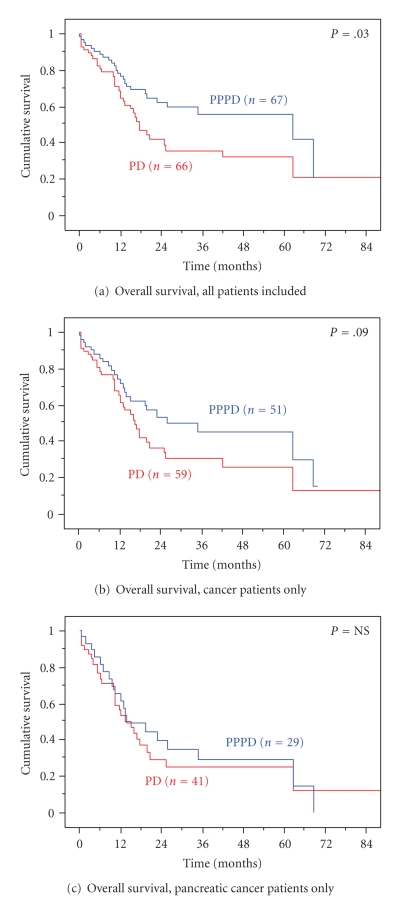
Overall actuarial survival, by resection group.

At a median follow-up of 15 months (20 for survivors), the overall actuarial survival is statistically superior in the PPPD group compared to PD patients by univariate analysis; the median survival time was 26 months after PPPD, and 16 months after PD (*P* = .03, Figure 4(a)). This certainly is confounded by the higher percentage of cancer, a greater frequency of pancreatic cancer, and a larger tumor size in the PD group. Consequently, the overall survival for cancer patients only (Figure 4(b)), or for individuals specifically with pancreatic cancer (Figure 4(c)) was not statistically different between the two- procedure groups.

## 4. DISCUSSION

In this series of 133 patients undergoing pancreatoduodenectomy within a single-surgeon practice, PPPD has been performed with constant criteria of intraoperative assessment, preservation of pyloric vagal innervation, and a duodenal lumen-protective anastomotic technique with dual-layer absorbable suture material. In addition, all duodenojejunostomies and gastrojejunostomies were placed as antecolic anastomoses. Our analysis of these prospectively collected data shows no obvious detriments to the use of PPPD, but also fails to show clear benefits. A PP procedure had been performed in half of the patients; it was associated with less complex operations based on reduced operative time, less intraoperative blood loss and fewer transfusions. However, there were no outcome differences between PD and PPPD groups regarding length of stay or cancer-specific survival. These apparent differences between the two procedures need to be interpreted thoughtfully, because some adverse clinicopathologic features appear to predispose a patient to the PD group. Importantly, there was no evidence to suggest that PPPDs are associated with a higher rate of postoperative delayed gastric emptying, or a greater NGT reinsertion need. Overall, the rate of DGE appears low in this series in both groups, and with the stated criteria for performing a PPPD, the outcomes relating to DGE shown here would certainly support its use.

The results of our study are consistent with larger randomized controlled trials and meta-analyses comparing intraoperative and postoperative outcomes between PPPD and PD. Our observed PPPD-associated reduction in average total operative time by 1.2 hours, in mean estimated blood loss by 223 mL, in transfusion rate by 20%, and in average PRBC number transfused by 0.31 generally correspond to numbers in part reported by others [8, 12, 13]. While it has to be remembered that these meta-analyses are largely based on the same trials, some of these randomized trials support similar intraoperative benefits of PPPD [9, 14] while others do not [11]. While the reduced operative time is intuitive, it is not sensible to assume that the mere addition of a distal gastrectomy should lead to significantly larger blood loss, transfusion rate, and possibly margin positivity. In our nonrandomized series, these findings are best explained with the higher percentage of pancreatic cancer cases in the PD group, where more challenging dissections tend to be encountered at the mesenteric vasculature. In our experience, this is the technical component that governs blood loss as well as margin status. When subgroups are well balanced regarding pancreatic versus other periampullary cancers, no differences in blood loss, and a comparable low R1 rate have been reported [10].

DGE is a concern that has been raised repeatedly in conjunction with the pylorus preserving procedure [4, 14, 20]. The cause of DGE is uncertain, and may involve factors that are either specific to the PP technique, or common to both PPPD and PD such as the loss of pancreatic polypeptide production associated with pancreatic head resection [7]. Experimental evidence suggests that preservation of the right gastric artery and the neurovascular bundle supplying the pylorus are critical to avoid DGE [21]. Our preferred technique of PPPD makes a strong attempt to preserve the right gastric artery and the tissue containing the pyloric branch of the vagus nerve, and we feel this may indeed contribute to the low rate of DGE in our study. Other factors specific to a PP approach may relate to technical aspects of the duodenal anastomosis, as this may easily be rendered too narrow through conventional stapling techniques. Interestingly, both patients in our series with DGE after PPPD had widely patent anastomoses, too, as documented via unimpaired radiographic emptying of intragastric contrast. This would rather suggest a functional disturbance of gastric motility than an anatomic obstacle, if DGE is still encountered after using the technique described. An antecolic duodenal reconstruction may be an important determinant to reduce the risk for DGE. As reported by Hartel et al., in a series of 100 patients undergoing PPPD, DGE occurred with significantly greater frequency after retrocolic anastomoses than antecolic reconstructions [22]. This notion is supported by a recent randomized prospective trial with 40 patients, although the resulting NGT duration of 4 versus 19 days, and the hospital stay of 28 versus 48 days are not applicable to a Western patient series [23]. A prospective evaluation of 50 patients with antecolic PPPD had demonstrated a DGE rate of 12%, higher than in our experience, but based on a different definition [24]. Importantly, DGE under these conditions was linked to the presence of other postoperative complications, including pancreatic leaks. Thus, the position of the duodenal anastomosis in relation to the transverse colon apparently is more important in determining DGE than how the pancreatic reconstruction is being performed [25]. Finally, DGE may be induced by a disturbance in intestinal splanchnic innervation after more radical resections, as suggested by a higher DGE rate after PD with extended retroperitoneal dissection compared to the lesser dissection group with a high PPPD number [10]. We do not perform a circumferential dissection of the superior mesenteric artery to avoid such morbidity.

Operative radicality in oncologic terms does not seem to be negatively affected by performing PPPDs. While a minimum number of 10 to 15 lymph nodes has been suggested to optimize staging accuracy and associated survival for both pancreatic and other periampullary cancers [26, 27], more radical resections have failed to improve survival of pancreatic cancer [10, 28, 29]. As our findings regarding lymph node counts suggest, this “radicality” is not affected by the PPPD modification. Positive margin resection is a powerful predictor for increased survival hazards, which also does not seem to be influenced by the performance of a PP [30]. In our series, the R0 resection rate was higher after PPPD, reflecting the fact that more aggressive malignancies with greater risks for radial margin involvement tend to require resection of antrum and pylorus based on intraoperative assessment. In this context, it should also be noted that volume-outcome relationships regarding both early postoperative mortality and long-term survival have been well demonstrated for pancreatic cancer, and that “standard” results as pertaining to PP procedures have to be carefully judged under these aspects as well [31, 32]. Virtually, all reports on PPPD and PD comparisons reflect high-volume institutions and cannot easily be translated into low-volume settings. Comparable morbidity and survival between PPPD and PD as seen in our results and those of others [11, 33] suggest that the standard PD is certainly an acceptable procedure for the treatment of periampullary malignancies. Subtle implications such as the possible need for continued medication to control gastric acid after PP (which we do not use) would easily sway our perceived balance in favor of a PD with antrectomy.

In conclusion, we report that PPPD is as effective as PD in resections for pancreatic or other periampullary cancers, provided the pylorus itself and the surrounding soft tissues are free from disease. Intraoperative advantages to PPPD are confounded in this series, but still of potential relevance. An oncologically appropriate procedure with acceptable early and late postoperative outcomes can be achieved with our PPPD approach. Although it remains uncertain whether pyloric vagal preservation, duodenal anastomotic technique, or antecolic reconstruction contribute decisively to the low rate of postoperative DGE and other morbidity events, we plan to continue their use within the described technique whenever judicious pre- and intra-operative assessment based on individual patient and disease factors supports a PPPD indication.

## Figures and Tables

**Table 1:** Demographic and pathologic data.

Demographic		Total cohort	PPPD	PD	*P* value
Patients (*n*)		133	67	66	N/A
Gender (%)	Female	59	66	52	NS
	Male	41	34	48	
Age, median (range) (years)		66 (39–88)	66 (45–88)	66 (39–86)	NS
ASA group 3 or greater (%)		59	59	60	NS
Diagnostic group (%)	Malignant	83	76	89	.04
	Benign	17	24	11	
Cancer type (% of patients with cancer)	Pancreatic	64	56	72	NS
	Ampullary	17	25	11	
	Other	18	19	17	
Tumor size, mean (cm)		3.1	3.8	3.5	.01
T3+ (%)		74	73	75	NS
N pos. (%)		62	55	68	NS
Grade 3+		82	84	78	NS
Total LN count, mean (*n*)		14.3	13.4	15.3	NS
R0 rate (%)		77	83	71	NS

**Table 2:** Operative treatment characteristics.

	Total cohort (*n* = 133)	PPPD (*n* = 67)	PD (*n* = 66)	*P* value
OR time (hour)	6.4	5.8	7	<.0001
EBL (mL)	527	413	636	.006
IVF (mL)	7381	6768	7954	.02
Transfusion rate (%)	23	13	33	.007
Units PRBC, mean	0.56	0.45	0.76	NS
Units PRBC per transfused patient (*n*)	2.2	2.2	2.2	NS
Urine output per hour operating time, mean (mL)	141	173	118	NS

**Table 3:** Postoperative outcomes.

	Total cohort (*n* = 133)	PPPD (*n* = 67)	PD (*n* = 66)	*P* value
Morbidity (%)	39	39	40	NS
Grade 3+ morbidity (%)	14	13	14	NS
Pancreatic leak (%)	11	12	11	NS
Lethal events (*n*)	6	2	4	NS
Median NGT duration (days)	1	1	2	NS
NGT reinsertion (%)	19	21	17	NS
Delayed gastric emptying (%)	3.8	3.0	4.5	NS
Delayed gastric emptying, international study group definition (%)	6.0	6.0	6.1	NS
Length of stay, median (d)	10	10	10	NS
